# 2341. Response to Vaccination Against COVID-19 in a Community Oncology Practice: a Rational for Antibody Testing

**DOI:** 10.1093/ofid/ofad500.1963

**Published:** 2023-11-27

**Authors:** Goar Egoryan, Guillermo Rodriguez-Nava

**Affiliations:** Ascension Health Saint Francis Hospital, Evanston, Illinois; Stanford University School of Medicine, Palo Alto, California

## Abstract

**Background:**

Patients with cancer are at increased risk of morbidity and mortality from COVID-19. Vaccination is crucial to prevent this potentially fatal infection. The aim of this study was to evaluate the seroconversion rate in response to 3 different COVID-19 vaccines in patients with cancer in a community setting.

**Methods:**

In this retrospective cohort study, we included 136 patients of a community oncology clinic with solid and hematological malignancies, fully vaccinated against SARS-CoV-2 and without a history of COVID-19. We evaluated qualitative seroconversion rates against the SARS-CoV-2 spike protein after 2 doses and after a booster dose, and the factors associated with increased risk for lack of seroconversion.

Cancer types in studied population

**Figure:**
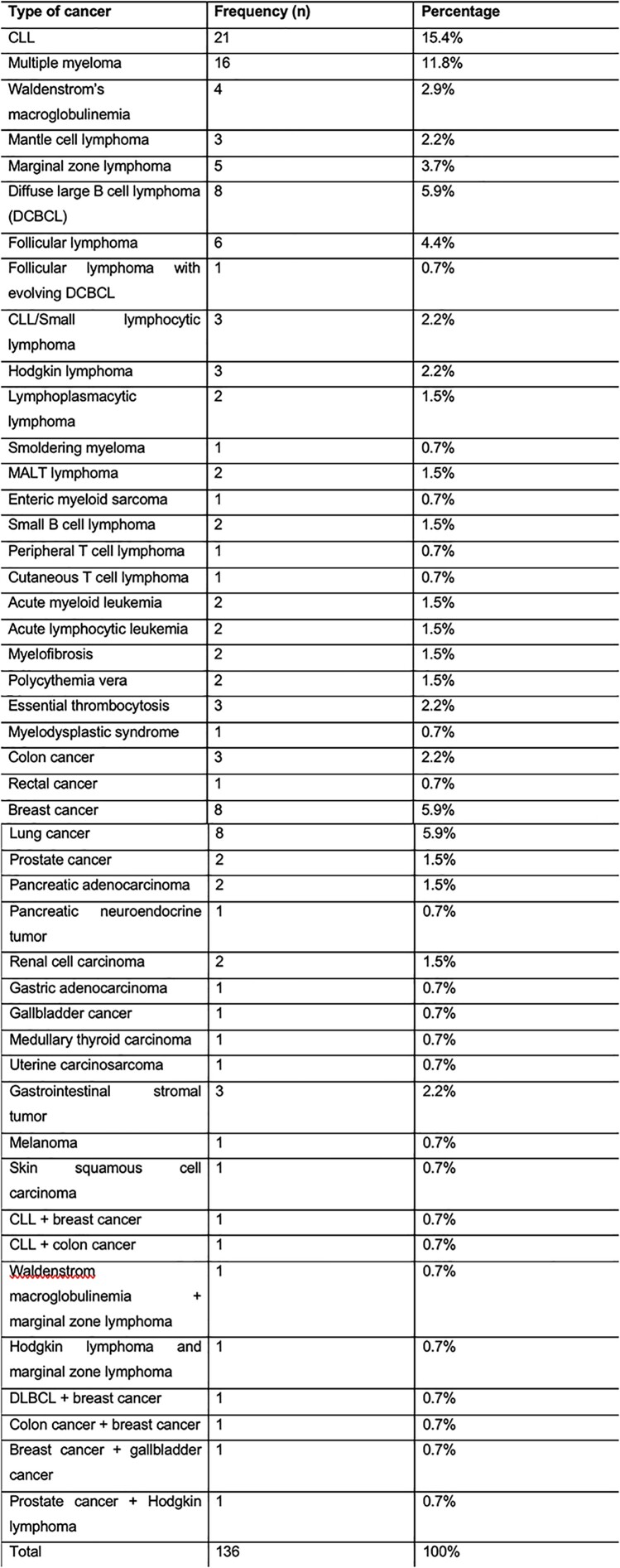

**Results:**

The median age was 71.5 years (IQR, 64 – 79.75 years), 67 (49.3%) were receiving active treatment; 94 (69.1%) patients had a hematological malignancy, 38 (27.9%) had a solid malignancy, and 4 (2.9%) had both. Only 90 (66.2%) patients developed antibodies against the SARS-CoV-2 spike protein after initial vaccination. 53.8% of those who did not initially develop antibodies, converted after a booster dose. In a multivariable binary logistic regression, patients with hematologic malignancies were nine times more likely to not develop antibodies compared to patients with a solid malignancy or both, when controlling for age, type of COVID-19 vaccine, and cancer therapy (odds ratio [OR] 9.57; 95% confidence interval [CI] 2.65 – 34.60, p=.001). Older age was also associated with an increased risk for not developing antibodies (OR 1.07; 95% CI 1.03 – 1.11, p < .001).

Antibody response after initial vaccination in each therapy group

**Figure:**
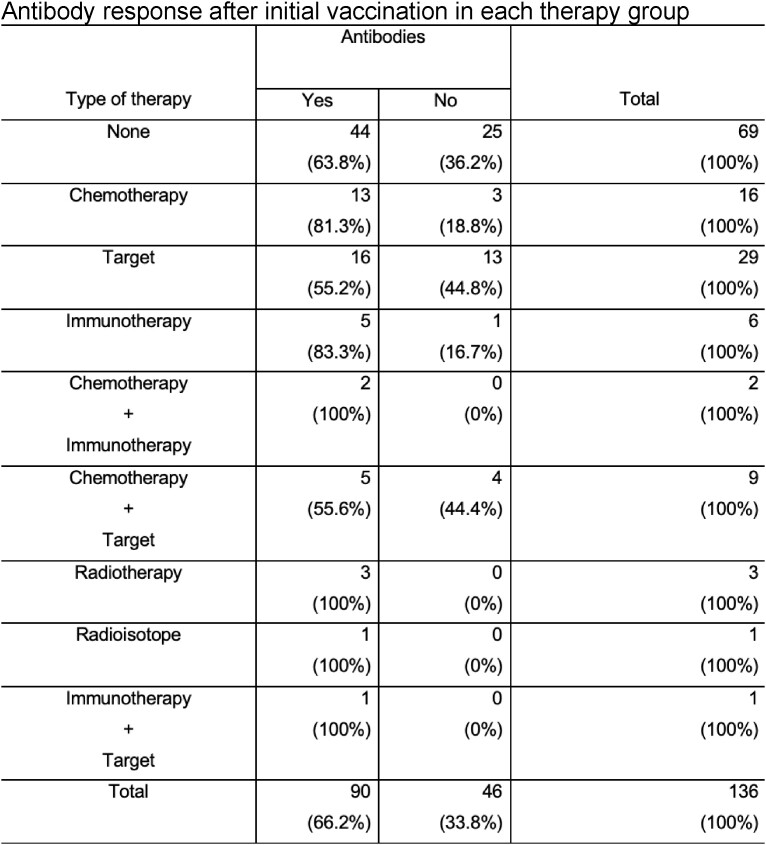

The rate of antibody production per each vaccine group (initial vaccination)

**Figure:**
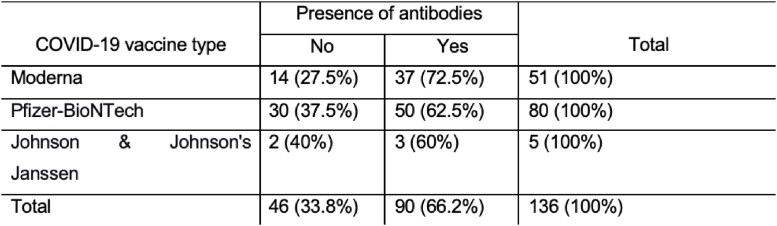

The rate of antibody production in response to booster per each hematologic malignancy type

**Figure:**
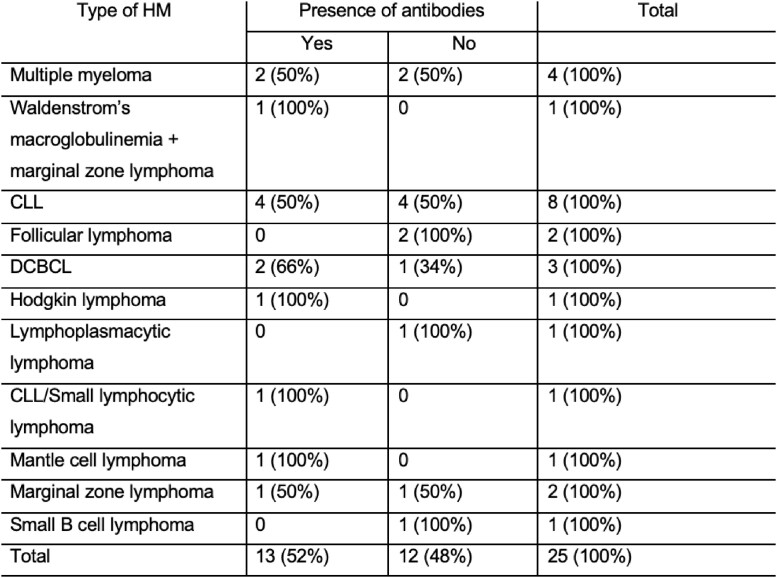

**Conclusion:**

In this population of patients from a community oncology clinic, older patients and patients with a hematological malignancy had an increased risk for not developing antibodies against the SARS-CoV-2 spike protein. A booster dose induced a response in a significant number of patients.

**Disclosures:**

**All Authors**: No reported disclosures

